# Impact of Different Styles of Online Course Videos on Students' Attention During the COVID-19 Pandemic

**DOI:** 10.3389/fpubh.2022.858780

**Published:** 2022-04-08

**Authors:** Qi Gao, Ying Tan

**Affiliations:** ^1^School of Economics, School of Marxism, Nankai University, Tianjin, China; ^2^College of Artificial Intelligence, Nankai University, Tianjin, China

**Keywords:** COVID-19, EEG, classroom attention, sample entropy, Mayer's theories of multimedia technology

## Abstract

**Background:**

The COVID-19 pandemic interfered with normal campus life, resulting in the need for the course to be conducted in an ideal online format. The purpose of this study is to analyze the impact of different styles of online political course videos on students' attention during the COVID-19 pandemic.

**Methods:**

Four college students participated in this small sample study. They were required to conduct two sessions of the experiment, in which they were required to watch three different styles of course videos in each session. While watching the videos, their EEG signals were acquired. For the acquired EEG signals, the sample entropy (SampEn) features were extracted. On the other hand, Mayer's theories of multimedia technology provide guidance for teachers' online courses to enhance students' attention levels. The results of EEG signals analysis and Mayer's theories of multimedia technology were combined to compare and analyze the effects of three styles of instructional videos.

**Results:**

Based on comparisons of the SampEn and Mayer's theories of multimedia technology analysis, the results suggest that online instruction in a style where the instructor and content appear on the screen at the same time and the instructor points out the location of the content as it is explained is more likely to elicit higher levels of students' attention.

**Conclusions:**

During the COVID-19 pandemic, online instructional methods have an impact on students' classroom attention. It is essential for teachers to design online instructional methods based on students' classroom attention levels and some multimedia instructional techniques to improve students' learning efficiency.

## 1. Introduction

At the end of 2019, due to the influence of the COVID-19 pandemic, mainly students study styles were changed greatly (1–3). Many schools were closed, and the students were unable to have a class as before (4). In order to solve the dilemma between epidemic prevention and teaching, schools started online courses (5, 6). Different from offline courses, students cannot face to face with teachers. How to organize the online courses and how to give the online courses in an appropriate format should be investigated (4, 7–10).

Classroom attention of students is related to the design of online teaching schemes (11). Different styles of online teaching styles can have an impact on students' attention levels (12). The attention level of students in the classroom is an important indicator to evaluate the effectiveness of teaching and learning. In addition, it is a prerequisite for students to maintain their attention in the learning process to learn effectively (13). Therefore, teachers can analyze students' classroom attention to explore more effective online teaching schemes, which can help students to improve their attention in online courses and improve their learning efficiency (14).

Attention can be monitored by physiological signals, such as electroencephalogram (EEG) signals (15). EEG signals can truly reflect the attentional state of students and can reflect the state of brain activity. Therefore, EEG signals are often used in studies to analyze attentional states. In addition, EEG acquisition devices are wearable devices, which are beneficial to be promoted and applied to detect students' classroom attention in actual teaching to assist teachers in better teaching activities (16). Entropy provides a way to quantify system regularity (17). In previous studies, Shannon entropy, dispersion entropy, multiscale entropy, approximate entropy, and sample entropy (SampEn) have been investigated as features to study the level of consciousness or attention-related EEG signals (18). In the study of Dawi et al. (19), Attention Deficit Hyperactivity Disorder patients have lower attention and smaller SampEn of EEG signals compared to normal individuals. To evaluate the level of attention in different states, Li et al. designed experiments with attentional, non-attentional and resting states, and the experimental results show that the SampEn is higher during the attentional state than that during the non-attentional state (20). Thus, previous studies have shown that the level of attention is proportional to the SampEn of EEG signals. In the study of Thomas et al. (21), EEG features based on SampEn were used to assess the attention level of participants in the game.

Online instruction relies on multimedia and an appropriate online multimedia instructional scheme which can reduce the external cognitive processing of learners and can make full use of the limited human cognitive capacity to help learners actively engage in cognitive processing (22). It is a key factor in maintaining a high attention level and enhancing learning efficiency for students during the COVID-19 pandemic. According to cognitive load theory (23) and working memory theory (24), there is a limited capacity of each channel in the human information processing system, and the cognitive resources of learners are required to be allocated during learning and problem-solving. The amount of cognitive processing that can occur in the verbal channel or the visual channel at any one time is extremely limited. Considering the characteristics of cognitive load and working memory, Mayer's theories of multimedia technology suggest how to present learners with verbal and picture information in the teaching process. Hence, two types of information will be processed in different information processing channels respectively (25). In this way, the cognitive resources of the learners are fully utilized, and thus the learners can better understand the knowledge and maintain their attention. For example, in multimedia instruction that follows the principle of temporal proximity, the picture and the narration are presented simultaneously (26). At this time, verbal processing and visual processing are carried out in separate information processing channels, which can reduce the cognitive load and facilitate learners' learning. In addition, including cues for learners on how to select and organize material can help learners reduce unnecessary extraneous cognitive processing and focus more attention on key elements, i.e., the signaling principle (27). Furthermore, according to the personalization principle (28), by using conversational voice to express verbal information in multimedia teaching, the learners can reap a better learning experience. The voice principle and the image principle as extensions of the personality principle (29, 30), using the human voice and agent to teach as if the learners are having a conversation, and both are viable ways to give learners a stronger sense of social presence.

**Figure 1:**
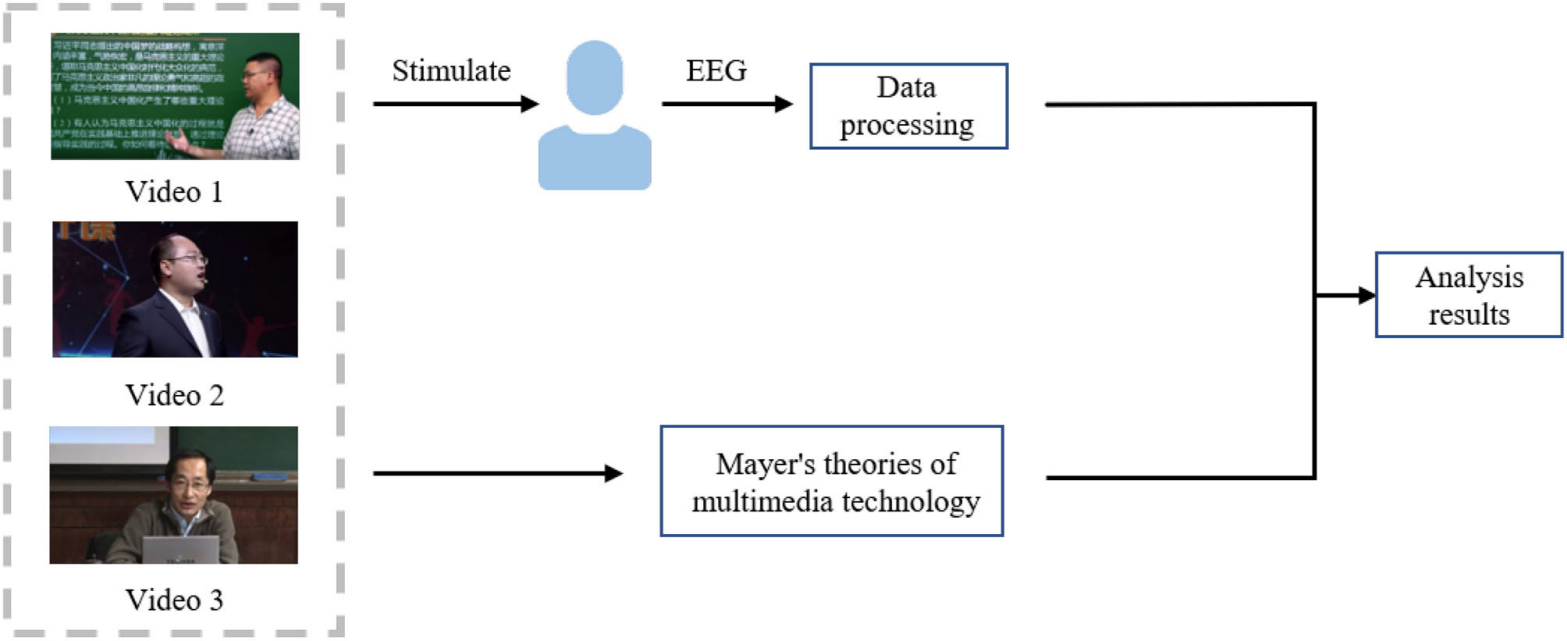
The framework of our work.

In this work, to investigate the effects of three styles of online instructional videos on students' attention levels, we conducted the analysis based on EEG signals and Mayer's theories of multimedia technology. The framework of the study is shown in Figure 1. The main contributions of our work are as follows:

- In order to obtain the real attentional state of the subjects, we acquired the EEG signals of the subjects while they were performing online learning for subsequent analysis.
- To analyze the level of attention during the experiment, we calculated the SampEn of the EEG signal as an evaluation index.
- In order to analyze the three styles of online instructional videos from multiple perspectives, we combined the results of SampEn calculations and Mayer's theories of multimedia technology for discussion.

This work is organized as follows. Section 2 introduces the experimental setup and data processing methods in our work. Sections 3 and 4 represent the results and discussion of the experiment, respectively. And the conclusions are summarized in section 5.

## 2. Materials and Methods

### 2.1. Experimental Setup

There were three instructional videos for our experiment. They were first matched with a number by random sorting. According to the sorting result, we named them as video 1, video 2, and video 3, respectively. And they were played following the numbered sequence from 1 to 3 in the experiment for all subjects. The three videos are different in styles and instructors. In video 1, the instructor and the content of the course appear simultaneously in the picture, distributed on both sides of the picture. Video 2 shows an instructor with many camera cuts. And in video 3, the instructor and the content appear alternately, and content appears in the key part. For the content, the three videos are both related to college political classes. Their knowledge points are different, but the topics and difficulties are similar.

**Figure 2:**
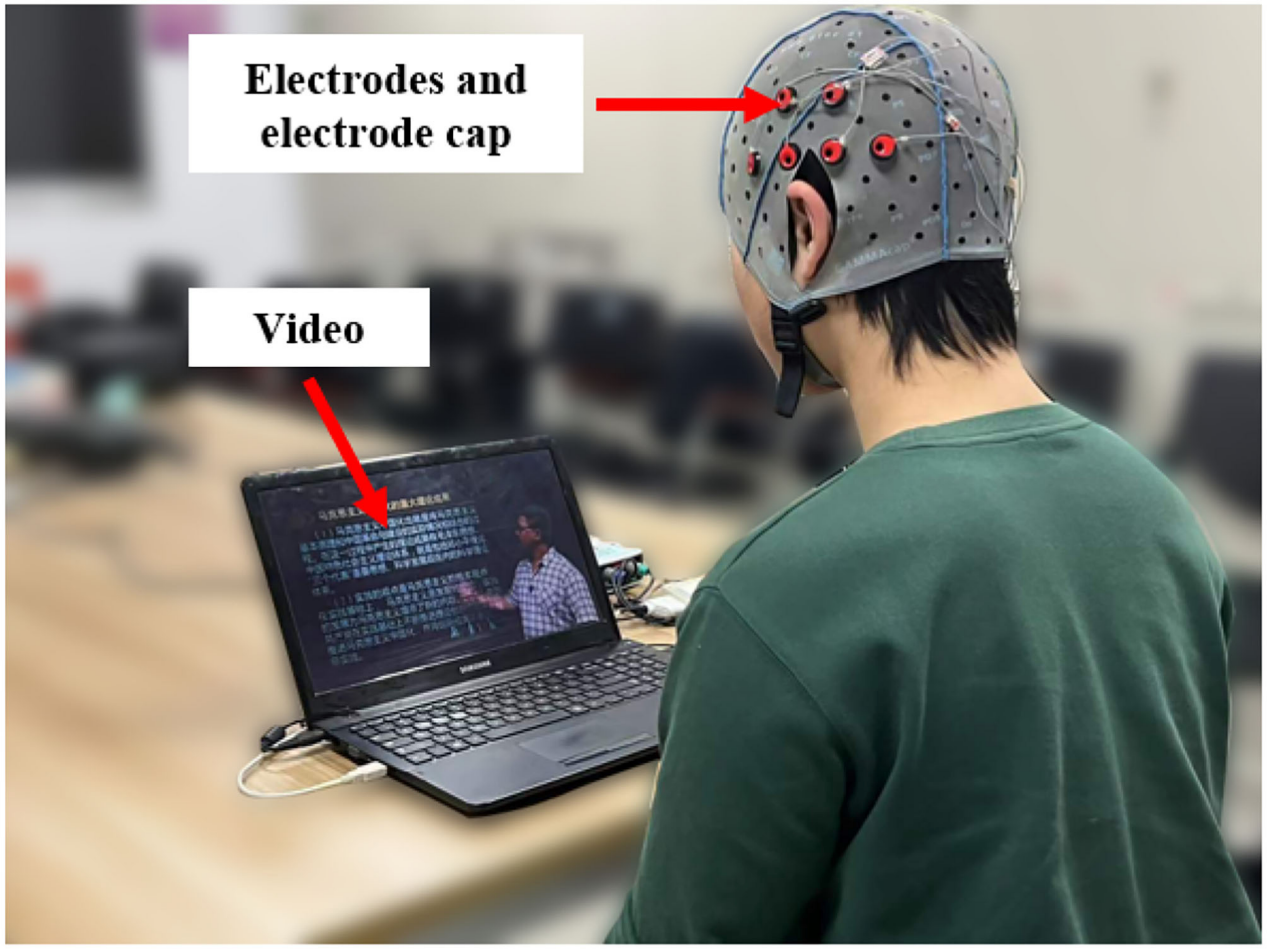
Schematic diagram of the experimental environment.

**Figure 3:**
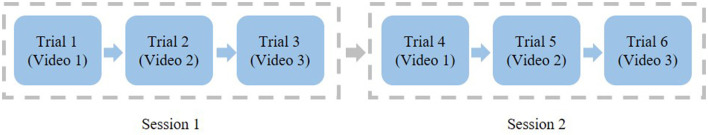
The experimental procedure for a subject.

**Figure 4:**
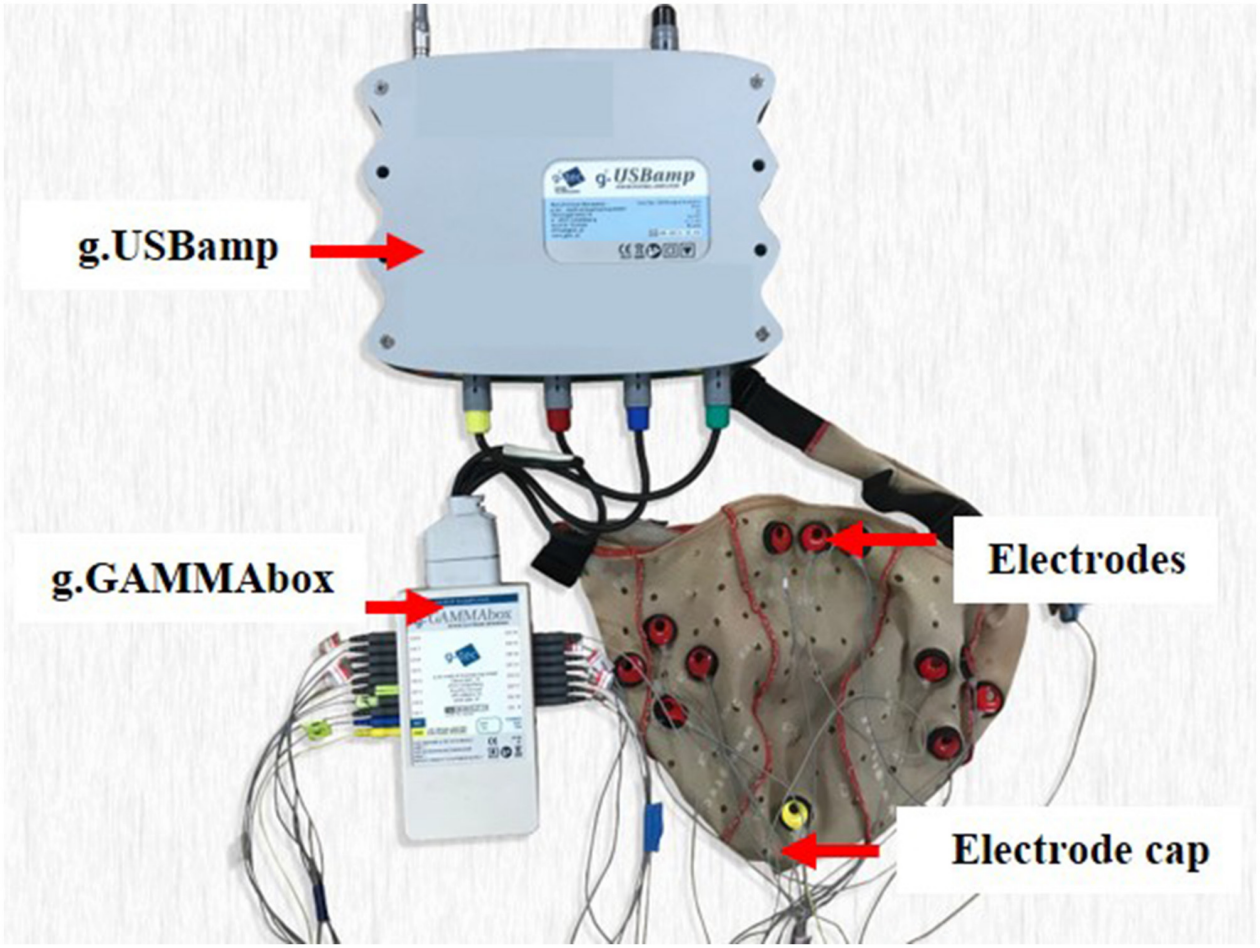
The equipment used to acquire the EEG data.

**Figure 5:**
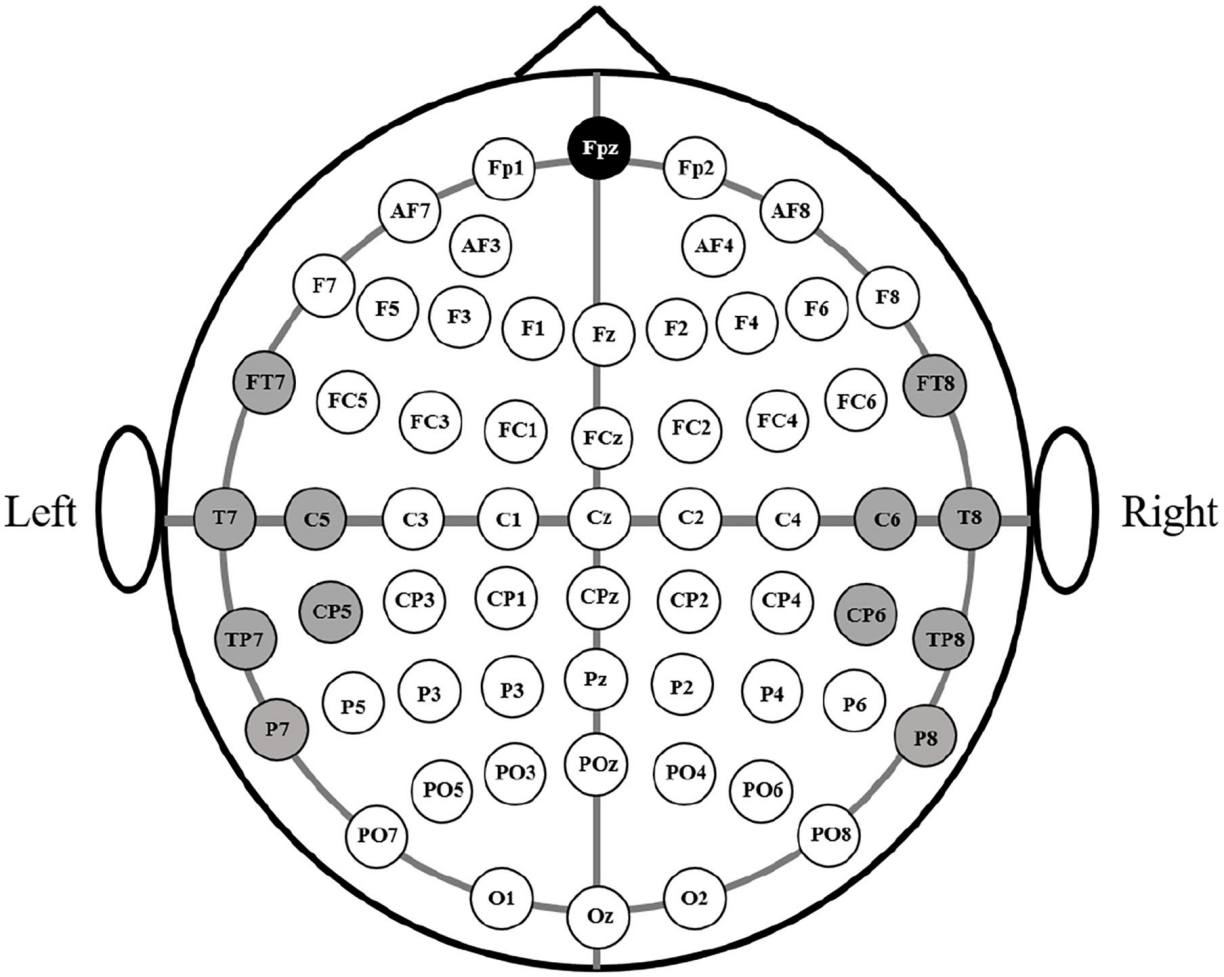
The positions of the electrodes according to the international 10–20 system.

Four healthy college students (3 males and 1 female) took part in the experiment. Their mean age was 27, ranging from 26 to 28. Figure 2 presents the experimental environment and the subjects were required to sit in front of the screen to watch the videos. The sound level and luminosity of the videos were appropriate for the subject that they felt comfortable with them of the video. The experiment was divided into two sessions and the experimental procedure is shown in Figure 3. There were three trials in a session. In one trial, the subject watched one video for approximately 5 min and their EEG signals were acquired at the same time. And each trial is separated by 1 min. The EEG signals acquisition equipment is shown in Figure 4. And EEG signals sampling frequency is 256 Hz. According to previous studies (31, 32) and the International 10-20 system, 12 electrodes (FT7, FT8, T7, T8, C5, C6, TP7, TP8, CP5, CP6, P7, P8) were used and their positions are shown in Figure 5.

### 2.2. Data Processing

The Butterworth filter is used to filter the EEG signal to the range of 1–50 Hz to eliminate high frequency noise and improve the signal-to-noise ratio. SampEn can be used to measure the complexity of a time series (33, 34). The higher the complexity of a time series is, the higher the SampEn it has. Conversely, time series with higher self-similarity has smaller SampEn. SampEn is independent of the length of the time series. And it is noise-resistant and stable, which is suitable for deterministic and random signals. Therefore, it is suitable for the analysis of EEG signals.

To calculate SampEn, two parameters need to be specified first: run length *m* and tolerance window *r*. For a given time series {*u*(*i*)} with *N* data points, e.g., EEG data with *N* sampling points, its SampEn can be calculated by the following steps:

Firstly, form the sequence {*u*(*i*)} into *m*-dimensional vectors *X*_*m*_(1) ...*X*_*m*_(*N*−*m*+1) in order. These vectors can be defined as *X*_*m*_(*i*) = [*u*(*i*), *u*(*i*+1), ..., *u*(*i*+*m*−1)](1 ≤ *i* ≤ *N*−*m*+1), which indicate *m* consecutive values of *u* beginning from the *i*-th point.

Secondly, calculate the distance between the vector *X*(*i*) and the rest of the vectors *X*(*j*), which is defined as:


(1) $$\begin{array}{l} d\left[X_{m}\left(i\right),X_{m}\left(j\right)\right]=\underset{k=0,...,m-1}{\max}\left(\left|u\left(i+k\right)-u\left(j+k\right)\right|\right) \end{array}$$

Thirdly, count the number of *d*[*X*(*i*), *X*(*j*)] corresponding to each *i*(1 ≤ *i* ≤ *N*−*m*+1) that is less than the given threshold *r*, denoted as *B*_*i*_. The proportion between this number and the total number of vectors is calculated according to formula (2).


(2) $$\begin{array}{l} B_{i}^{m}\left(r\right)=\frac{1}{N-m-1}B_{i} \end{array}$$

And the average for all i is defined as formula (3), where *B*^*m*^(*r*) is the probability that two sequences match with m points.


(3) $$\begin{array}{l} B^{m}\left(r\right)=\frac{1}{N-m}\overset{N-m}{\underset{i=1}{\sum }}B_{i}^{m}\left(r\right) \end{array}$$

Then, change the dimension to *m*+1 and calculate *B*^*m*+1^(*r*), which is the probability that two sequences match *m*+1 points. Finally, formula (4) is used to calculate the SampEn of the sequence, denoted as *H*.


(4) $$\begin{array}{l} H\left(m,r\right)=\underset{N\to \infty }{\lim}\left\{-\ln\;\left[B^{m+1}\left(r\right)/B^{m}\left(r\right)\right]\right\} \end{array}$$

In this work, we set the parameters *m* and *r* to be 2 and 0.2σ, respectively, and σ is the standard deviation of the series.

### 2.3. Statistical Analysis

The Kruskal-Wallis test is a non-parametric test that is applied to test whether there is a statistically significant difference in the medians between three or more groups (35). It does not require the assumption that the data conform to a normal distribution. Another non-parametric test, the Mann-Whitney U test, can be used when there are only two groups of data, and it can be used to test for differences in means between data (36). In our work, we evaluated the impact of these videos on students' attention by testing whether the features were significantly different. We used the Kruskal-Wallis test to determine the significance of the difference in the effect of three videos in the same session on attention. In addition, the Mann-Whitney U test was used to determine the difference in student attention between the two sessions.

## 3. Results

**Figure 6:**
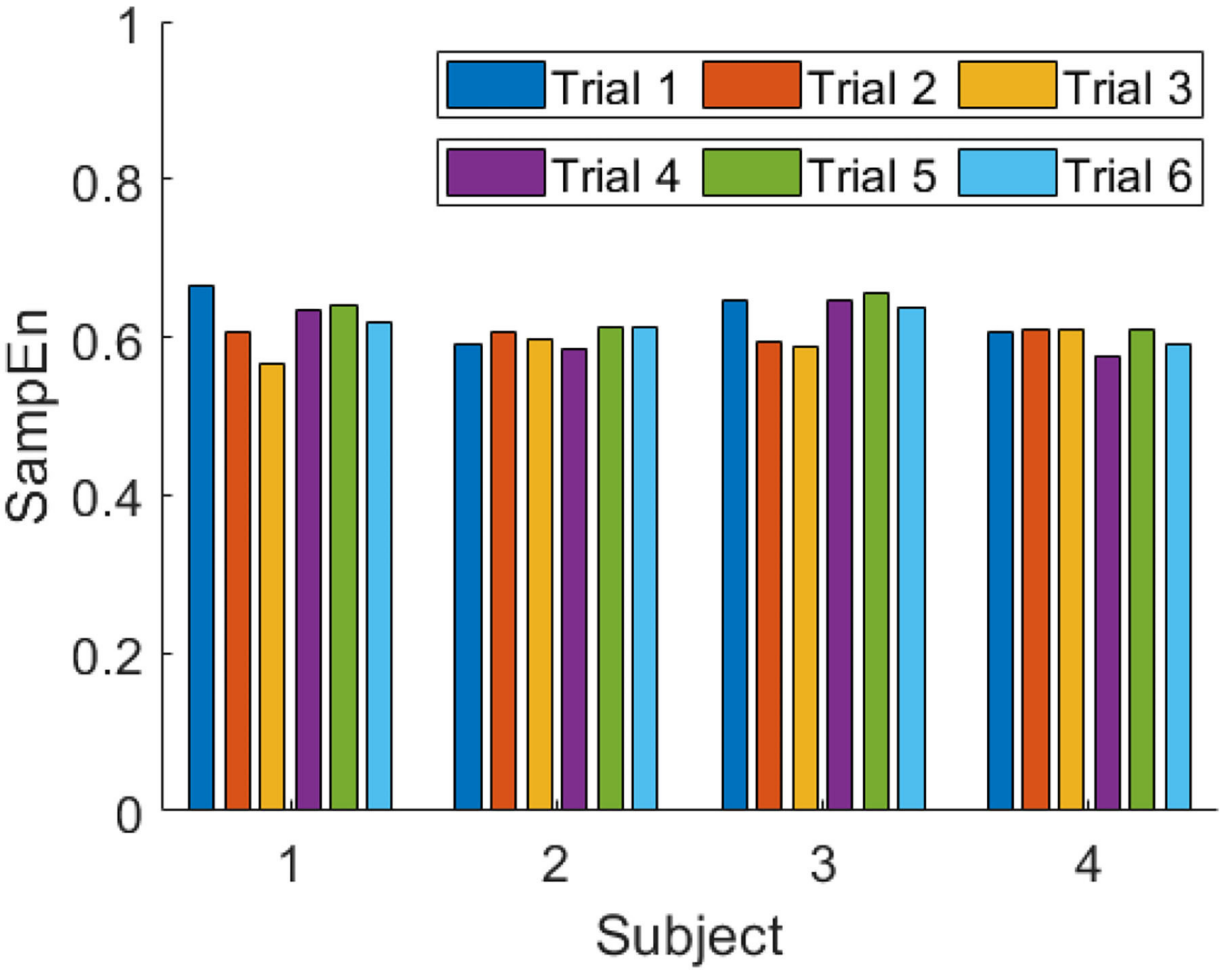
SampEn of all trials for each subject.

For each trial, the SampEn is calculated with non-overlapping 4s time windows. According to the results of previous studies, a higher SampEn represents a higher level of attention. In Figure 6 and Table 1, it is shown that the SampEn of each trial for each subject is the mean value of the SampEn for each time window. By comparing the SampEn of each video in the two sessions, we can see that the SampEn of video 1 in session 1 is higher than that in session 2, while that of video 2 and video 3 are higher in session 2.

**Table 1:** The values of SampEn for each subject.

	**SampEn**					

	**Video 1**		**Video 2**		**Video 3**	
**Subject**	**Trial 1**	**Trial 4**	**Trial 2**	**Trial 5**	**Trial 3**	**Trial 6**
1	0.6640	0.6328	0.6061	0.6398	0.5669	0.6189
2	0.5899	0.5863	0.6062	0.6134	0.5975	0.6123
3	0.6459	0.6469	0.5928	0.6564	0.5889	0.6368
4	0.6071	0.5758	0.6104	0.6088	0.6108	0.5906
Average	0.6268	0.6104	0.6039	0.6296	0.5910	0.6146

**Figure 7:**
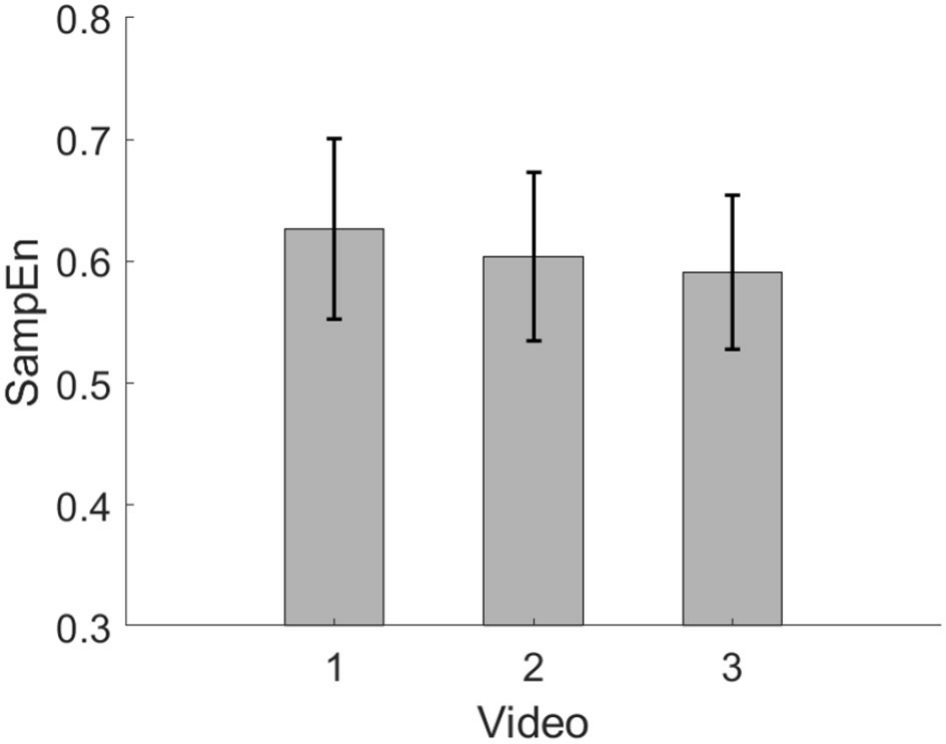
Average SampEn of three videos in session 1.

**Figure 8:**
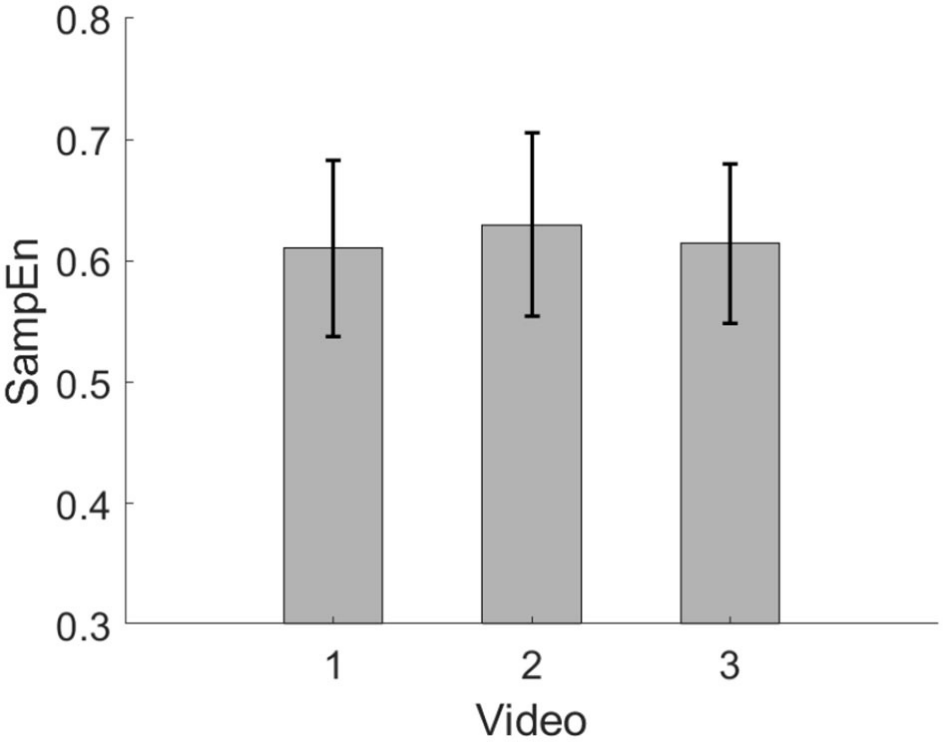
Average SampEn of three videos in session 2.

Figures 7, 8 show the average SampEn of the three videos in session 1 and session 2, respectively. In session 1, the SampEn of video 1 is the highest. In session 2, the SampEn of video 2 is the highest.

Table 2 presents the average of the SampEn of the EEG for all experiments for each subject. These results reflect individual variability and the effect of videos of different styles on attention. In addition, it is also reflected that there are different levels of attention for the same subject at different viewing times of the same video.

**Table 2:** Average SampEn for each subject.

**Subject**	**1**	**2**	**3**	**4**
Average SampEn	0.6214	0.6009	0.6279	0.6006

Table 3 shows the average of the SampEn of the three videos in all trials. From the results, it can be seen that video 1 stimulates higher attention levels of the subjects. In addition, video 2 and video 3 stimulate close levels of attention.

**Table 3:** Average SampEn of three videos.

**Video**	**1**	**2**	**3**
Average SampEn	0.6186	0.6167	0.6028

**Figure 9:**
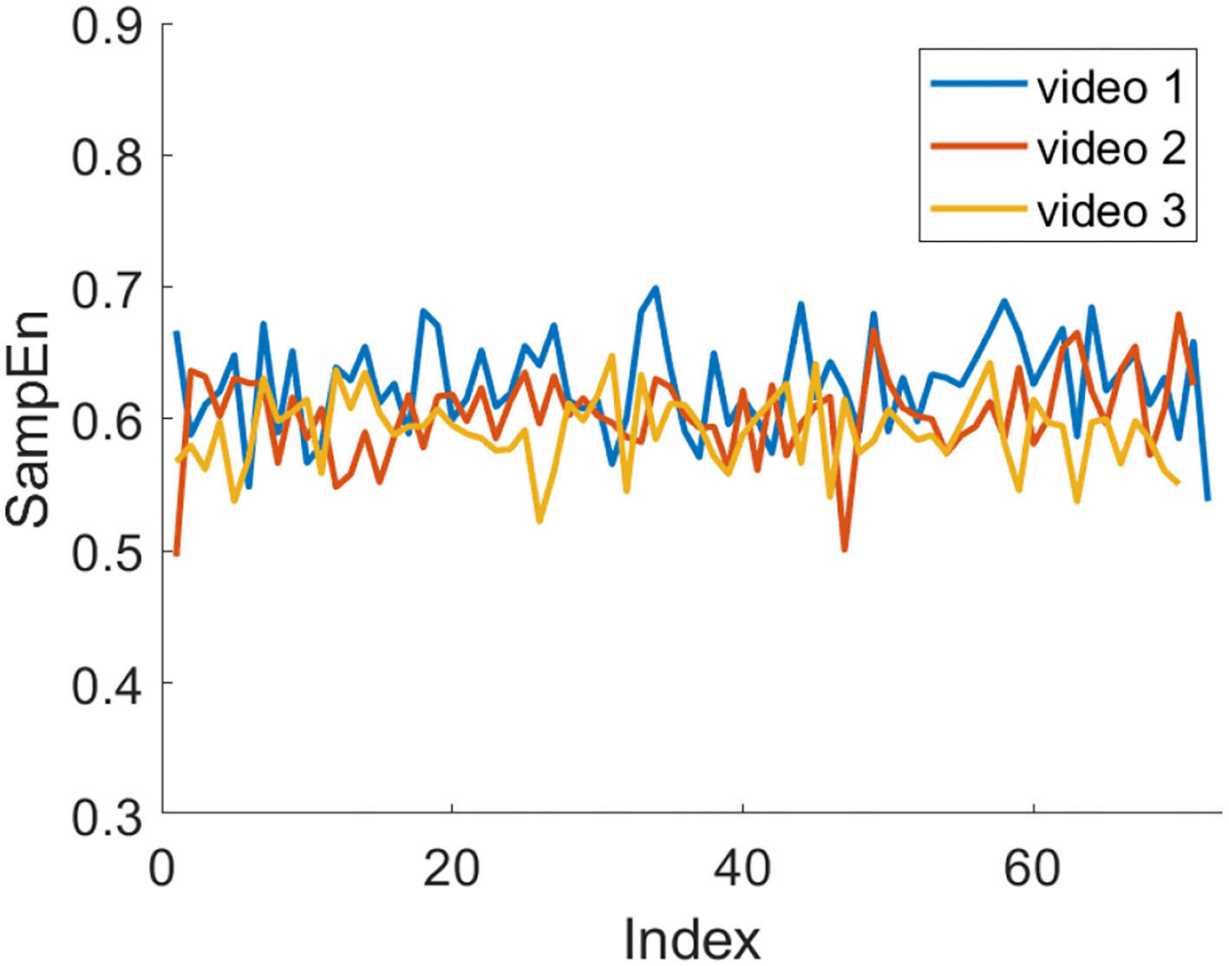
Average SampEn of three videos in session 1 over time.

**Figure 10:**
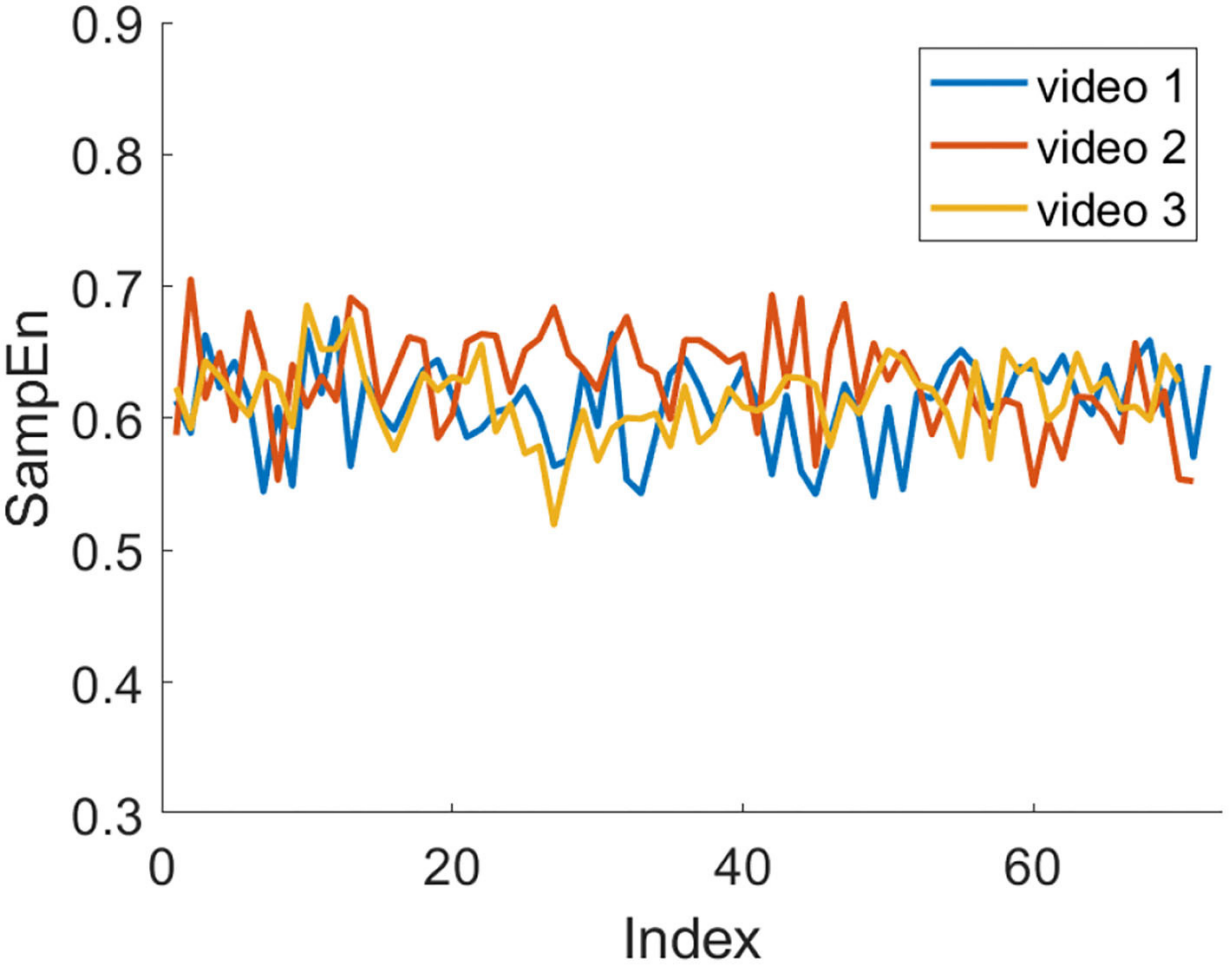
Average SampEn of three videos in session 2 over time.

Figures 9, 10 show the trend of the average SampEn of each video for all subjects in session 1 and session 2, respectively. In session 1, video 1 is higher than the other two videos most of the time. In session 2, video 2 is higher than the other two videos most of the time. Besides, it can be seen that the SampEn decreases or fluctuates more in the middle or later stages of video viewing.

Based on the average SampEn of each video for all subjects in session 1 and session 2, we evaluated the attentional impact using two non-parametric tests. Table 4 shows the Kruskal-Wallis test results of three trials in two sessions. The low *p*-values (*p* < 0.05) indicate that the difference in the impact of the three videos on students' attention was significant. Moreover, we determined the difference between the three videos in the two sessions and the Mann-Whitney U test results are presented in Table 5. The results illustrate that there is a significant difference in the impact of the same video on the subjects in the two sessions (*p* < 0.05).

**Table 4:** The Kruskal-Wallis test results of three trials in two sessions.

**Session**	**1**	**2**
*p*-value	9.35 ×10^−9^	4.07 ×10^−3^

**Table 5:** The Mann-Whitney U test results of three videos.

**Video**	**1**	**2**	**3**
*p*-value	1.38 ×10^−2^	3.39 ×10^−5^	2.36 ×10^−6^

## 4. Discussion

Noticing that different styles of online instructional styles during the COVID-19 pandemic can have an impact on students' learning attention levels. In this work, the SampEn of EEG signals was used as an indicator of attention to assess the three styles of instructional videos, which is significant for teachers to organize and improve online teaching styles.

In terms of the mean SampEn analysis, the mean SampEn of the EEG signal corresponding to video 1 is 0.6186, which is higher than 0.6167 for video 2 and 0.6028 for video 3, i.e., video 1 is able to induce a higher level of attention. According to Mayer's theories of multimedia technology, video 1 conforms to the theory of image on the screen, i.e., adding the image of the narrator on the screen. At the same time, the narrator on the screen will point toward what is being taught with his or her hand. Such a style of instructional videos can enhance the subjects' social presence and make them feel like they are learning from a real person, which contributes to their attention level.

Based on the comparison between the before and after the two sessions, a possible explanation is that as video 1 is easy to understand, so subjects decreased their attention to video 1 in session 2, and increased their attention to video 2 and video 3 in session 2.

In terms of the analysis of the SampEn change for each video, the SampEn fluctuated more or tended to decrease in the middle and later stages of some trials. The possible reason for this phenomenon is that as the learning time progresses, the subjects become less focused and their attention level decreases. The decline in attention is a common phenomenon in the classroom, and this experimental phenomenon is consistent with the changing pattern of attention of classroom students. Furthermore, it is an online course, and the lack of direct communication and supervision in reality, as well as the influence of the surrounding environmental factors, can affect the subject's attention. Therefore, it is a feasible way for teachers to organize online instruction by incorporating guided activity, reflection, feedback, pacing, and pre-training to build an interactive multimedia learning environment (37), which is a practical solution to improve students' attention in class and enhance the quality of online instruction. Moreover, the individual variability can be taken into account so that as many students as possible can achieve meaningful learning in the classroom.

This work is a preliminary study and still has some limitations that require further research in the future. Firstly, in our work, we selected videos with similar topics as stimuli to minimize the effect of content differences on students' attention. However, it is better to have the videos with exactly the same content. Therefore, in order to reduce the effects of other factors, it is necessary to use materials with the same content to perform future experiments. Moreover, to better analyze the impact of multiple styles of online instruction on students' classroom attention, there is a need for us to design more complete experimental paradigms in the future by adding experiments on resting, non-attentive, and attentive states. It is also to obtain labels of the data for more accurate analysis. At the same time, we need to add more subjects to provide a more comprehensive and generalized basis for online multimedia instructional design.

## 5. Conclusions

The COVID-19 pandemic brings negative effects to schools, and it is difficult for students to enjoy courses with teachers face to face. In this circumstance, online courses are flourishing in many schools, and students can take the courses at home during the COVID-19 pandemic. To improve the effect of online courses, this work analyzed the impact of different styles of online instructional videos on the classroom attention of the subjects. We collected the EEG signals of the subjects while watching these online course videos and calculated the SampEn of the EEG signals as a measure of attention. And video 1 has the highest average SampEn. Combined with Mayer's theories of multimedia technology, the results show that online course videos in which the instructor and the content appear on the screen at the same time and the instructor points out the location of the content while explaining it are more likely to induce higher levels of students' attention. And it is a useful basis for online multimedia instruction design in the COVID-19 pandemic.

## Data Availability Statement

The raw data supporting the conclusions of this article will be made available by the authors, without undue reservation.

## Ethics Statement

Ethical review and approval was not required for the study on human participants in accordance with the local legislation and institutional requirements. The patients/participants provided their written informed consent to participate in this study.

## Author Contributions

QG designed the framework and acquired the data. YT acquired and analyzed the data. QG and YT wrote the manuscript. Both authors contributed to the article and approved the submitted version.

## Funding

This work was supported in part by Key Project of Special Task Project of Scientific Research Plan of Municipal Education Commission in 2021 (Mental Health Education) under Grant 2021ZDGX01.

## Conflict of Interest

The authors declare that the research was conducted in the absence of any commercial or financial relationships that could be construed as a potential conflict of interest.

## Publisher's Note

All claims expressed in this article are solely those of the authors and do not necessarily represent those of their affiliated organizations, or those of the publisher, the editors and the reviewers. Any product that may be evaluated in this article, or claim that may be made by its manufacturer, is not guaranteed or endorsed by the publisher.
